# Steroid Production and Follicular Development of Neonatal Mouse Ovary during *in vitro* Culture

**Published:** 2013-09-18

**Authors:** Shabnam Abdi, Mojdeh Salehnia, Saman Hosseinkhani

**Affiliations:** 1 Department of Anatomy, Faculty of Medical Sciences, Tarbiat Modares University, Tehran, Iran; 2 Department of Biochemistry, Faculty of Biological Sciences, Tarbiat Modares University, Tehran, Iran

**Keywords:** *in vitro*, Primordial Follicles, Ovary, Organ Culture

## Abstract

**Background:**

The aim of this study was to investigate follicular growth and steroid production in neonatal mouse ovary during *in vitro* culture.

**Materials and Methods:**

In this experimental study, 7-day-old mouse whole ovaries were cultured in α-MEM (medium supplemented with 100 mIU/ml recombinant follicle stimulating hormone, 1% insulin, transferrin and selenium (ITS), 5% fetal bovine serum (FBS), 100 IU/ml penicillin and 50 μg/ml streptomycin for 7 days. The size of whole ovary was determined as mean area during culture. The survival rates of isolated preantral follicles after culture were assessed using trypan blue staining after being mechanically isolated. Histological evaluation of whole ovary was done by hematoxylin and eosin staining. 17-β estradiol, progesterone and dehydroepiandrosterone concentrations in the medium were measured during culture.

**Results:**

The mean area of ovary increased after culture (1.47 vs. 0.21 mm^2^). The survival rate of isolated follicles in ovary after culture was 99.2%. There was a significant decline in the percentage of primordial follicles after seven days of culture (91.8 ± 0.2% vs. 65.1 ± 1.1%), whereas the rate of preantral follicles increased significantly (4.6 ± 0.4% vs. 29.2 ± 0.5%). The levels of estradiol, progesterone and dehydroepiandrosterone also increased significantly after culture (p<0.001).

**Conclusion:**

These results show that the growth and development of primordial follicles in contrast with hormonal production decreased during *in vitro* culture of neonatal mouse ovaries.

## Introduction

Mammalian ovarian tissues contain a large number of resting pool of primordial follicles, which represent the reproductive potential of females. However, the number of follicles is limited and also gradually reduced during the female reproductive life (1, 2). Therefore, a considerably interesting issue in reproductive biology is how to initiate growth of these follicles to improve reproductive potential. In vivo and *in vitro* follicular growth and development are two alternative methods to initiate and activate their development within whole ovaries (3-6).

Almost 70 years ago techniques were used by Martinovitch (7) for *in vitro* culture of mammalian whole ovaries. Since then, *in vitro* organ or fragment culture has been evaluated under different culture conditions in several species, such as mice (6, 8-11), cattle (12, 13), baboon (14), bovine (15) and human (16, 17). Eppig and O’Brien in 1996 cultured newborn mouse ovaries for 8 days and activated primordial follicles and grew them to the secondary stage. The isolated secondary follicles were cultured for 14 days, and mature and fertilizable oocytes and one living offspring were obtained (6).

O’Brien et al. (8) reported the development of oocytes derived from *in vitro* cultured primordial follicles isolated from cultured ovarian fragments and 59 living offsprings were produced.

The key advantage of this type of culture of ovarian fragments or whole ovary is that it preserves normal interactions between follicles at different sizes and stages and various extra follicular cell types within the ovarian tissue. However, the main disadvantage of this technique is that in large tissue fragments culture, degeneration and necrosis of tissues occur due to inadequate oxygenation. However additional investigations are required to improve this culture system.

The objective of the present study was to establish a mouse ovarian organ culture system where it could be used to evaluate a variety of factors which participate in folliculogenesis. During this organ culture, morphological and hormonal assessments of cultured ovaries were done.

## Materials and Methods

### Animals and ovarian tissue

In this experimental study, one week (n=15) and two weeks (n=5) old female mice obtained from National Medical Research Institute (NMRI) were housed and bred in the Central Animal House of Tarbiat Modares University. All animals were housed under a 12-hour light/12-hour dark regime at 22-24˚C.

The mice were sacrificed by cervical dislocation and their ovaries were dissected free of fat and mesentery. For each one-week-old mouse, one ovary was selected randomly and fixed immediately in Bouin’s solution (non-cultured control) and the other was considered for *in vitro* culture. Both ovaries of two-week-old mice were considered as control and fixed in Bouin’s solution.

### Organ culture

Each ovary (n=15) was cultured in 24-well plates with tissue culture well inserts (non-tissue culture treated, PICM 012 50, 0.4-µm pore size; Millipore Corp, Billerica, MA) in 0.4 ml α- MEM (Gibco, UK) supplemented with 5% fetal bovine serum (FBS), 100 mIU/ml recombinant follicle stimulating hormone (rFSH or Gonal-f, Serono, Switzerland), 1% insulin, transferrin and selenium (ITS, Gibco, UK), 100 IU/ml penicillin and 50 μg/ ml streptomycin. Approximately 400 μl of culture medium was added to the compartment below the membrane insert, such that ovaries on the membrane were covered with a thin film of medium. The ovaries were incubated at 37˚C and 5% CO2 for 7 days. Every other day, 150 μl media was replaced with fresh culture medium. The collected media were stored separately at -80˚C until undertaking the hormonal assay.

### Histological evaluation

To assess the integrity of follicles after culturing, the follicular morphology was examined by histological staining. The non-cultured (control) ovaries from 7- and 14-day-old mice and cultured ovaries (for 7 days) from one-week-old mice were fixed in Bouin’s solution and were embedded in paraffin wax and serially sectioned at 5-μmthickness (n=5 for each group); every five sections of each ovary were mounted on glass slides, and stained with hematoxylin and eosin. All sections were examined using light microscopy at a magnification of ×400. For this study, stages of follicles have been classified and counted according to the method described previously

### Evaluation of the ovarian follicular viability using trypan blue

The survival rates of the isolated preantral follicles from cultured ovaries (n=30 in each group) were determined using trypan blue staining. The preantral follicles with 120 μm in diameter from the ovaries were mechanically isolated using insulin-gauge needles under stereomicroscope. Only follicles containing several layers of granulosa cells with a centrally located, healthy, visible oocyte and a thin layer of theca cells were selected. These isolated follicles were stained using trypan blue (0.4%) (Sigma, St. Louis, MO). The follicles were recorded as degenerated or survived: degenerated follicles stained blue and surviving ones not stained (13).

### Ovarian area

Photographs of each ovary in all groups of study were prepared under Olympus (Tokyo, Japan) CK40 inverted microscope with an attached Olympus DP11 digital camera. All photographs were imported into ImageJ 1.33U software (National Institutes of Health, USA). Area was calculated in units of pixels and then converted to millimeter based on the pixel number to millimeter conversion ratio determined by measurements using the calibrated millimeter.

### Hormonal assays

17-β estradiol (E2), progesterone (P4) and dehydroepiandrosterone (DHEA) were measured in collected media derived from ovarian culture during days 2, 4 and 6. The levels of 17-β estradiol (Monobind, USA, sensitivity=6.5 pg/mL) and progesterone (DiaPlus, USA, sensitivity=0.1 ng/ml) were measured by an enzyme immunoassay modified for the cell culture media. DHEA was measured with a commercial immunoenzymatic assay using antibodies directed against the α-subunit (Monobind, USA, sensitivity=20 pg/ml). These experiments were at minimum done in triplicates.

### Statistical analysis

Statistical analysis was done with SPSS 16.0 software. The ovarian area and production of hormones were compared by one-way analysis of variance (ANOVA) and Tukey’s test. Student’s ttest was used to compare the proportion of follicular stages. P<0.05 was considered to be statistically significant.

## Results

### Ovarian morphology

**Fig 1:**
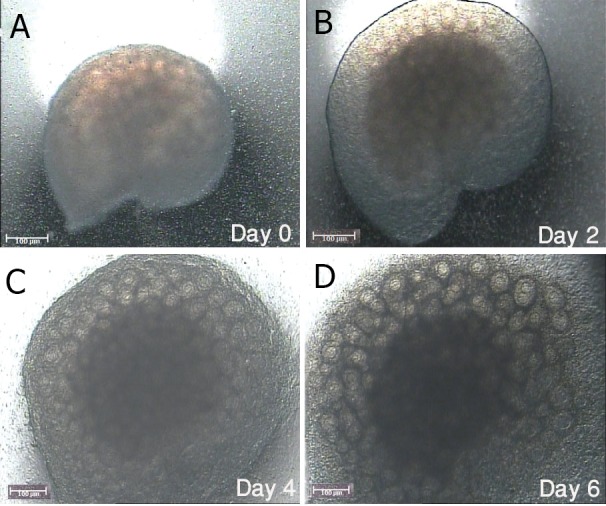
Photomicrographs of 7-day-old mouse ovary viewed under the invert microscope in non-cultured fresh samples (A) and during one week of culture (B-D).

**Fig 2:**
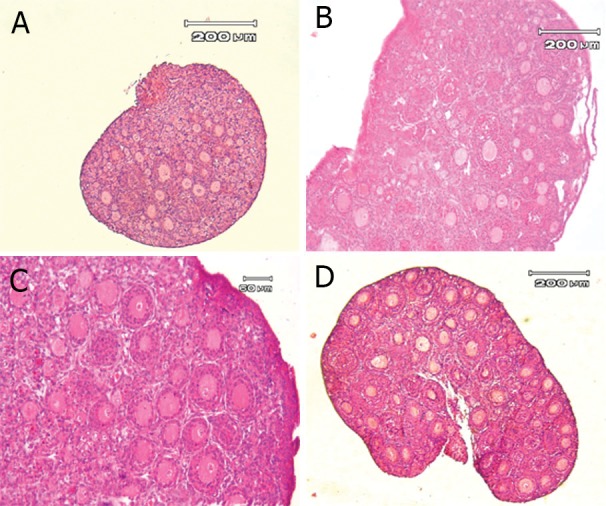
Hematoxylin and eosin staining of fresh and cultured mouse ovarian organ. Non-cultured fresh one week old mouse ovary which contains mainly primordial follicles with a few primary and secondary follicles (A), one week old mouse ovary after 7 days of culture (B). Higher magnification of one week old mouse ovary after 7 days, more secondary follicles were observed (C). Non-cultured fresh 14-day-old mouse ovary (D).

The phase contrast morphology of ovaries is shown in fig 1. The appearance of growing follicles became apparent during the culture period and could be observed as swellings on the surface of the cultured ovary. The morphology of ovaries derived from 7-dayold mice before culture (non-cultured control) using the hematoxylin and eosin staining are shown in fig 2A, after one week culture in figs 2B and 2C and the ovaries of two-week-old mice in fig 2D. This figure showed that the structural organization of cultured ovaries was well preserved and appeared normal and it was similar to two-week-old mouse ovaries. Furthermore the necrosis area with atretic follicles (with a retracted oocyte and granulosa cells, pyknotic nucleus) was consistently seen in the central part of the cultured ovary

### Normality rate of follicles

**Fig 3:**
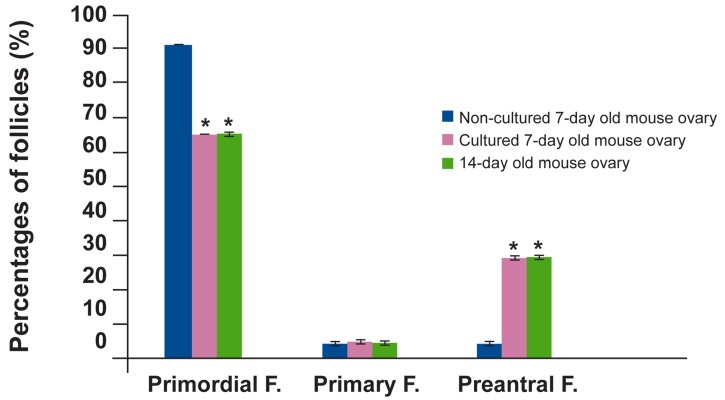
Normality rates of follicles after 7 days of *in vitro* ovarian culture. *; significantly different with one week non-cultured ovary.

The normality rates of follicles at various developmental stages within the ovaries of cultured and non-cultured controls are presented in fig 3. Ovaries derived from 7-day-old mice at the beginning of the culture contained mostly primordial follicles (91.8 ± 0.2%), with a small proportion of primary (5.8 ± 2.5%) and secondary follicles (4.6 ± 0.4%). After 7 days of *in vitro* organ culture, primordial follicles represented a smaller percentage of the total follicle pool (65.1 ± 1.1%), similar to the follicle distribution seen in ovaries from 14-day-old mice (65.2 ± 0.8%). The proportion of preantral follicles increased significantly during 7 days of culture, from 4.6 ± 0.4% to 29.2 ± 0.5% (p<0.001). 

### The viability of isolated follicles

**Fig 4:**
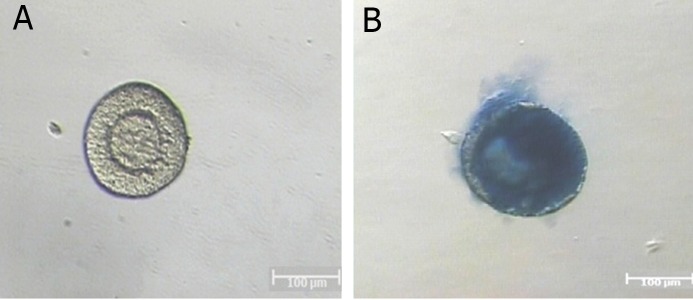
The trypan blue staining of preantral follicles. A. The survived follicle was not stained and B. degenerated follicle was stained intensively.

The morphologies of preantral follicles after trypan blue staining are shown in fig 4. The survival rates of preantral follicles derived from cultured ovaries using negative trypan blue staining was 99.2% and this rate was 100% in the control group of 14-day-old mice ovaries. There was no significant difference between them (p>0.05).

### 

**Fig 5:**
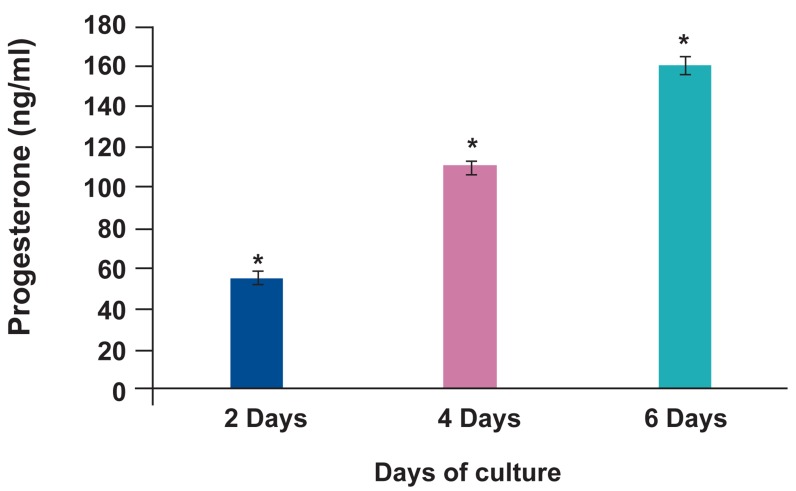
Area of mouse ovary during 7 days of culture. *; There were significant differences between lengths of culture

Area of cultured ovaries The area of cultured ovaries increased significantly from 0.212 ± 0.05 mm^2^ on day 0 to 1.47 ± 0.1 mm^2^ on day 7 of culture (p<0.003, Fig 5).

### Hormonal assay

**Fig 6:**
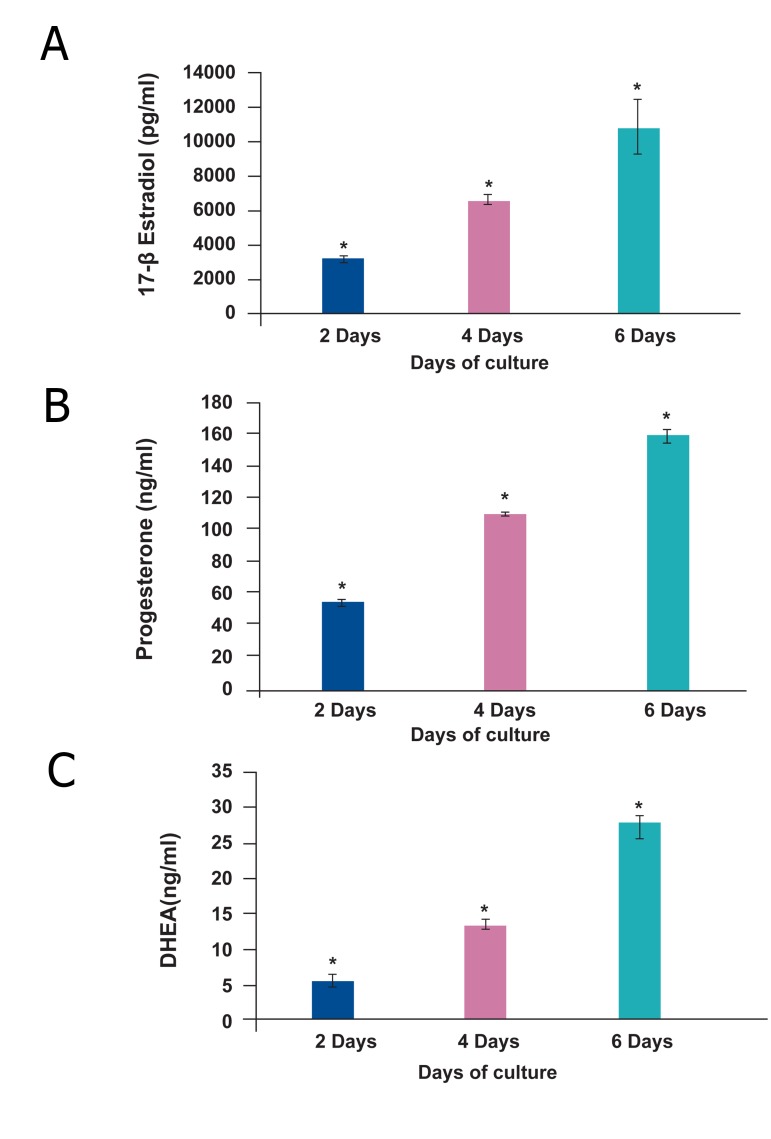
The level of estradiol (A), progesterone (B) and dehydroepiandrosterone (C) in collected culture media of mouse ovary. *; There were significant differences between lengths of culture.

The levels of E2, P4 and DHEA during different lengths of culture are compared in fig 6. The levels of E2 in culture media on day 2, 4 and 6 were 3381 ± 43, 6552 ± 214 and 12938 ± 684 pg/ml and the concentration of P4 on the same days were 55.7 ± 0.5, 111 ± 3 and 157 ± 14 ng/ml respectively. DHEA levels increased from day 2 to day 4 to day 6 of culture (6.8 ± 0.2, 14.5 ± 0.2 and 29 ± 0.5 ng/ml respectively).

## Discussion

The establishment of a successful culture system for primordial follicle growth and development is essential to studies of *in vitro* follicle and ovarian culture and these kinds of studies are only at the incipient and nebulous stage.

Data obtained from this histological study showed that neonatal mouse ovary has a high percentage of primordial follicles. However, the proportion of these follicles decreased after 7 days of *in vitro* culture to a level observed in a 14-day-old mouse ovary. Concurrently, there was a sustained increase in the number of preantral follicles from day 2 to day 6 of culture.

Our observation confirmed that the ovarian culture conditions used in this study had a significant effect on follicular activation and growth. The culture of mouse whole ovary in α-MEM medium supplemented with 10% FBS and 1% ITS not only increased the number of large follicles but also the ovarian area, implicating that an increase in ovarian area during culture may represent follicular growth and development.

In comparison with a previous work on the culture of isolated follicles (18), the whole ovary culture seems to yield better results. There are three possible explanations for the observed results.

First, *in vitro* ovarian culture provided follicles with an in situ growth environment resembling the ovary in vivo (19). The maintenance of the follicle architecture supports the critical cellular interactions between adjacent somatic cells and between somatic and germ cells, which preserves local biochemical control pathways that trigger the initiation of folliclular growth. In similar results, Jin SY et al. (20) demonstrated that culture of 8-day-old mouse ovaries for 4 days resulted in transition of the follicle population from primordial and primary to secondary follicles. These studies firstly establish that the activation of primordial follicles occur spontaneously, without the addition of growth factors or hormones. Second, the ovarian culture on tissue culture inserts covered by a thin film of medium survived better than isolated follicles. Third, enzymatic or mechanical dissociation of small follicles may have harmful effects on the subsequent follicular development (18).

Ovarian steroid production is an indicator of ovarian development and function. During the primordial follicle transition to the preantral follicle, granulosa and theca cells synthesize estradiol and androstenedione progressively, therefore measuring E2, P4 and DHEA could be an appropriate tool to evaluate the functionality of these cells within the ovary. The measurable steroidogenic function of these cells may be due to the remarkable increase in proliferation and differentiation of follicle cells and aromatase activities in granulosa cells during culture (21, 22)

## Conclusion

Results of this study show that *in vitro* ovarian organ culture could induce growth and development of follicles and steroid production, which indicate normal physiologic endocrine function of ovarian follicles during culture. Moreover ovarian culture alone is not able to complete the full growth and development of follicles to potentiate the competence of oocytes for fertilization. Thus, further research is needed to study *in vitro* culture of isolated follicles from ovarian culture. This study brings science one step closer to enhanced *in vitro* follicle culture methods, which may ultimately lead to clinical application in fertility treatments.
